# Cognitive, Affective Problems and Renal Cross Ectopy in a Patient with 48,XXYY/47,XYY Syndrome

**DOI:** 10.1155/2015/950574

**Published:** 2015-05-05

**Authors:** Sefa Resim, Faruk Kucukdurmaz, Nazım Kankılıc, Ozlem Altunoren, Erkan Efe, Can Benlioglu

**Affiliations:** ^1^Department of Urology, Kahramanmaras Sutcu Imam University, Kahramanmaras, Turkey; ^2^Department of Urology, Nizip State Hospital, Gaziantep, Turkey; ^3^Department of Psychiatry, Kahramanmaras State Hospital, Kahramanmaras, Turkey; ^4^Department of Urology, Adiyaman University, Adiyaman, Turkey

## Abstract

Klinefelter syndrome is the most common sex chromosome abnormality (SCA) in infertile patients and 47,XXY genomic configuration constitutes most of the cases. However, additional Xs and/or Y such as 48,XXYY, 48,XXXY, and 47,XYY can occur less frequently than 47,XXY. Those configurations were considered as variants of Klinefelter syndrome. In this report, we present an infertile man with tall stature and decreased testicular volume. Semen analysis and hormonal evaluation supported the diagnosis of nonobstructive azoospermia. Genetic investigation demonstrated an abnormal male karyotype with two X chromosomes and two Y chromosomes consistent with 48,XXYY(17)/47,XYY (13). Additionally, the patient expressed cognitive and affective problems which were documented by psychomotor retardation and borderline intelligence measured by an IQ value between 70 and 80. Systemic evaluation also revealed cross ectopy and malrotation of the right kidney in the patient. The couple was referred to microtesticular sperm extraction (micro-TESE)/intracytoplasmic sperm injection (ICSI) cycles and preimplantation genetic diagnosis (PGD). To the best of our knowledge, this is the first report of combination of XYY and XXYY syndromes associated with cognitive, affective dysfunction and renal malrotation.

## 1. Introduction

Sex chromosomal aneuploidy (SCA) is the most common disorder of sex chromosomes in human, with an incidence of 1 in 400 newborns [1]. In Klinefelter syndrome, 47,XXY is the most common SCA, but additional Xs and/or Y such as 48,XXYY, 48,XXXY, and 47,XYY can occur less frequently than 47,XXY [2]. It was reported that the incidence of 48,XXXY and 48,XXYY is 1 in 17.000 and 1 in 50.000 male births, respectively. The frequency of 49,XXXYY karyotype is as rare as 1 in 85.000 to 100.000 boys [3]. Klinefelter syndrome was initially focused on endocrinologic and physical characteristics; however, psychiatric issues and social dysfunction were also reported in this disorder [2, 3]. The addition of more than one extra X and/or Y chromosome to a normal male karyotype is less frequent and has its own distinctive physical and behavioral profile [1–4]. The XXYY syndrome was previously accepted as a variant of Klinefelter syndrome with hypogenitalism disorders [5]. However, specific clinical features including mental retardation and psychiatric problems have been described. Some neurodevelopmental and psychological disorders are more common and significant in 48,XXYY, 48,XXXY, and 49,XXXXY syndromes and are typically more severe and/or complex when compared with 47,XXY. Developmental dyspraxia may have an effect on the early language and motor deficits [6, 7]. Attention deficit hyperactivity disorder (ADHD) is present in over 70% of males with 48,XXYY, with symptoms of inattention, distractibility, poor organizational skills, hyperactivity, and/or impulsivity affecting daily functioning [8]. This is significantly higher than the 35–45% rate of ADHD in 47,XXY. In addition to ADHD, autism spectrum disorders (ASD) were much more common in 48,XXYY subjects with a rate of 28–50% [8, 9]. The XYY karyotype is rarely diagnosed during childhood or even in the adult [10]. It is now well recognized that the majority of XYY males had normal phenotype; however, variable abnormalities including skeletal, cardiovascular, and genital systems and behavioral problems have been described in the literature and can lead to clinical suspicion of the XYY syndrome. While infertility, various congenital abnormalities, and psychiatric problems were described in XXYY and XYY syndromes, the presence of renal malrotation in 48,XXYY, 47,XYY has not been reported yet. We herein report an infertile man showing XYY/XXYY syndrome associated with cognitive, affective problems and malrotation and cross ectopy of the right kidney.

## 2. Patient

**Figure 1:**
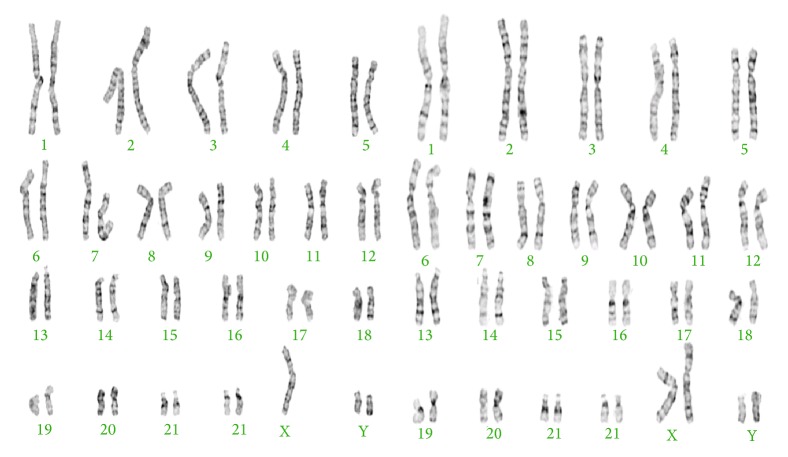
Karyotype of the patient.

**Figure 2:**
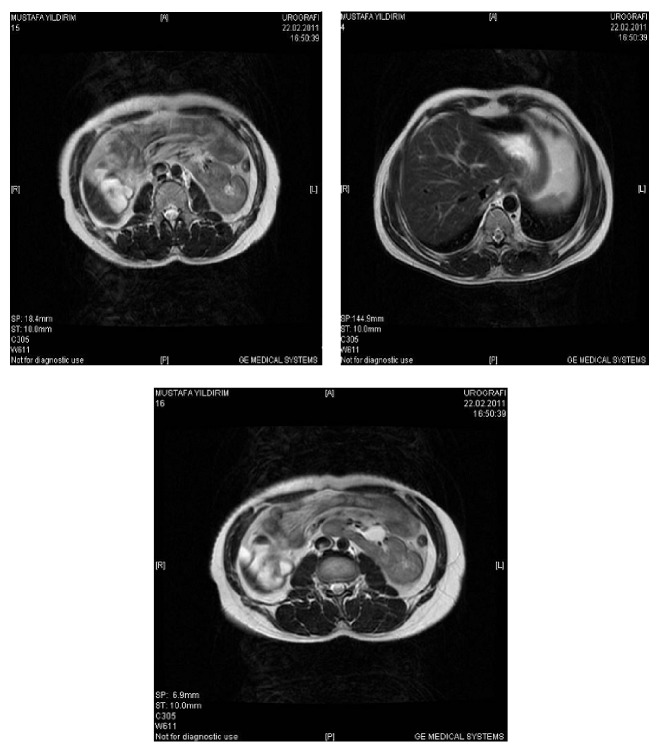
Abdominal CT showing cross ectopy and malrotation of the right kidney.

A couple was referred to our outpatient clinic with the diagnosis of primary infertility. He was 27 years old, illiterate, and unemployed. His physical examination revealed no abnormality except for a decreased testicular volume (12 mL for the right testicle and 8 mm for the left one) and tall stature (1.85 cm and 80 kg). His wife was 24 years old and had normal menstrual cycles. Her gynecological evaluation was also normal. The patient underwent repeated semen analysis, hormonal evaluation, karyotyping of peripheral blood lymphocytes, and molecular tests for Y chromosome microdeletions. Repeated semen analysis showed azoospermia (pellet negative). Hormonal measurements were made by radioimmunoassay. The patient had increased FSH levels (21.9 mUI/mL (normal level (*n*): 0.7–11.1 mUI/mL)), normal LH (8.73 mUI/mL (*n*: 0.8–7.6 mUI/mL)), and total testosterone level 474 ng/dL (*n*: 262–1593 ng/dL). No AZF microdeletion was detected by two different multiplex PCR methods. Chromosomal analysis of peripheral blood lymphocytes was conducted using the standard methods. The proband proved to be a carrier of chromosomal aneuploidy that is mos 48,XXYY(17)/ 47,XYY (13) as seen in Figure 1 [10]. His partner's karyotype was found to be normal. During the examination we determined that the patient expressed cognitive and affective problems with suspected psychomotor retardation. When he was evaluated with Kent EGY test [11] and Porteus Labyrinth test [12], he appeared to have borderline intelligence, with a documented IQ of 70–80. Patient was not able to finish the elementary school. There was no family history of psychiatric or genetic disorders. It was observed that the patient had prominent negative signs which were assessed by PANSS (Positive and Negative Syndrome Scale) [13]. Additionally, systemic evaluation of patient by computerized tomography of the abdomen revealed cross ectopy and malrotation of the right kidney (Figure 2). The couple was referred to an in vitro fertilization center for micro-TESE/ICSI cycles and PGD.

## 3. Discussion

Klinefelter syndrome presents as XXY in all body cells in 80% of cases and as mosaic (XY/XXY) in the other 20%. Rarely can multiple line mosaicisms be found. The parents' recurrence risk for another chromosomally abnormal live birth is 1% to 2%. The 48,XXYY aneuploidy, first described by Muldal et al. in 1960, was thought for a long time to present as a cytogenetic variant of the Klinefelter syndrome [14]. Most of 48,XXYY cases are thought to result from an aneuploid sperm produced through two consecutive nondisjunction events in both meiosis I and meiosis II in a chromosomally normal father, so a boy with XXYY has one X chromosome from his mother and the additional XYY from his father [15]. In 47,XYY syndrome, the extra Y chromosome results from paternal nondisjunction at meiosis II. Those patients are usually fertile, and their sperm cells mostly contain abnormal karyotype. It has been hypothesized that one of the two Y chromosomes is lost before entering spermatogonia in meiosis. Males with 48,XXYY karyotype display neurodevelopmental disorders (ADHD and ASD), mental retardation, aggressive behavioral problems, hypogonadism, small testes, gynecomastia, and increased rate of varicose veins [16–18]. Males with increased numbers of extra Xs or Ys are at risk of oral language and auditory processing problems that may place them at risk of learning deficits, emotional issues, and school and/or social adjustment difficulties [11]. Males with 48,XXXY have reduced cognitive functions when compared to 48,XXYY, since addition of each X decreases the overall IQ by 15-16 points. It is difficult to precise that these specific features occurred due to the extra X or Y chromosome in these disorders, since additional Y chromosomes are often accompanied by additional X chromosomes (48,XXYY, 49,XXXYY) [9]. The most common clinical features of 47,XYY patients are tall stature, reduced IQ, and poor motor coordination together with numerous nonspecific dysmorphic features associated with minor skeletal abnormalities such as radioulnar synostosis and congenital heart diseases [19]. In the present report, the patient was unable to finish elementary school and had borderline intelligence, with a documented IQ of 70–80. In addition, the patient had negative signs which were assessed by PANSS. Those findings were consistent with the literature. Visootsak et al. suggested that males with 48,XXYY are anxious and easily frustrated or impatient [3]. In a study of 16 males with 48,XXYY compared to 9 males with 47,XXY between the ages of 5 and 20, findings indicate that 48,XXYY males have verbal and full scale IQs significantly lower than males with 47,XXY [9]. 48,XXYY males are also prone to have problems with hyperactivity, aggression, conduct, and depression compared to males with 47,XXY. Furthermore, 48,XXYY males have significantly lower adaptive functioning than males with 47,XXY [9]. Semen analysis and hormonal measurements of the case provided the diagnosis nonobstructive azoospermia. Similar to other cases with 48,XXYY karyotype, our patient had tall stature and decreased testicular volume [6, 9]. Additionally, systemic evaluation of the patient revealed that he had cross ectopy and malrotation of the right kidney. Abdominal CT showed that the right kidney was located in the left retroperitoneal space over the left kidney and fused with the latter in a narrow area. A slight malrotation of the right kidney was also determined. Previous cases with renal aplasia and ectopy were described in 48,XXYY males; however, cross ectopy and malrotation of the kidney in 48,XXYY/47,XYY patients have not been reported in the literature yet [5, 20].

## 4. Conclusion

Genetic evaluation should be performed in all nonobstructive azoospermia patients. Our experience confirms that gonosomal aneuploidies with multiple X and Y chromosomes represent a distinct group of disorders that may be recognized at birth or during childhood in relation to their peculiar phenotype and neuropsychiatric profiles. However, the diagnosis of 48,XXYY/47,XYY may be delayed to adulthood. Since various skeletal, cardiac, and renal anomalies associated with this syndrome were reported, systemic evaluation should be performed in all patients.
